# Supplementary material for: Proteomic and functional analyses of the periodic membrane skeleton in neurons
Source: Nat Commun. 2022 Jun 9;13:3196. doi: 10.1038/s41467-022-30720-x (PMC9184744; doi:10.1038/s41467-022-30720-x)
Supplement: Supplementary file 1 — Supplementary Information [file 41467_2022_30720_MOESM1_ESM.pdf]

Supplementary Information for

Proteomic and functional analyses of the periodic membrane skeleton in neurons

Ruobo Zhou*, Boran Han, Roberta Nowak, Yunzhe Lu, Evan Heller, Chenglong Xia, Athar H. Chishti, Velia M. Fowler, Xiaowei Zhuang*

* To whom correspondence may be addressed. Email: zhuang@chemistry.harvard.edu and ruobo.zhou@psu.edu

This PDF file includes:

Supplementary Figures

Supplementary Reference

Supplementary figures

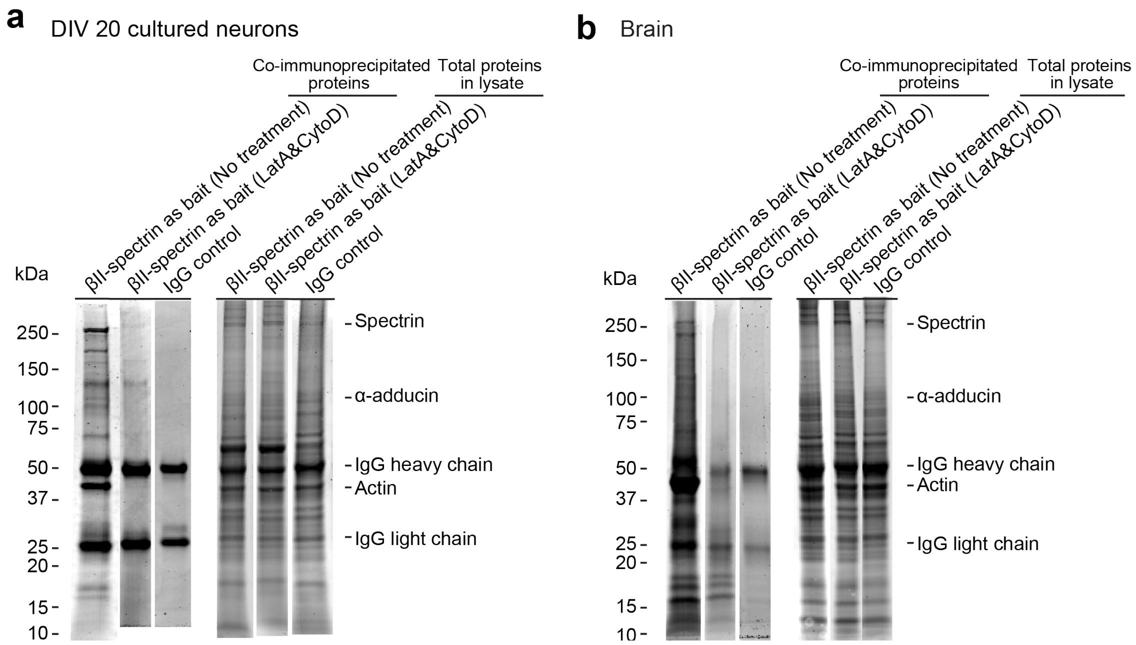

Supplementary Figure 1: SDS-PAGE images of co-immunoprecipitated proteins from cultured mouse hippocampal neurons and the adult mouse whole brain. a SYPRO Red-stained SDS-PAGE images of co-immunoprecipitated proteins from cultured mouse hippocampal neurons (DIV 20) using the βII-spectrin antibody (left most lane) and an irrelevant antibody IgG (an antibody that is not directed against any known antigen) as the control (third left lane). As an additional control, 20 μM LatA and 40 μM CytoD were used to disrupt the MPS (second left lane). Also shown are the corresponding SYPRO Red-stained SDS-PAGE images of total cell proteins in the lysates from the mouse hippocampal neurons (right three lanes). The labels next to the gel images indicate the positions for the expected weights of spectrin (~280 kDa), α-adduin (~103 kDa), actin (~42 kDa), IgG heavy chain (HC, ~50 kDa), and IgG light chain (LC, ~ 25 kDa). **b** Same as (**a**) but for the adult mouse whole brain instead of the cultured mouse hippocampal neurons (DIV 20). The input protein amount is ~2 mg for the co-IP experiments using the cultured mouse hippocampal neurons (DIV 20), or ~10 mg for the co-IP experiments using the adult mouse whole brain. The gel images are representative examples from two independent experiments with similar results.

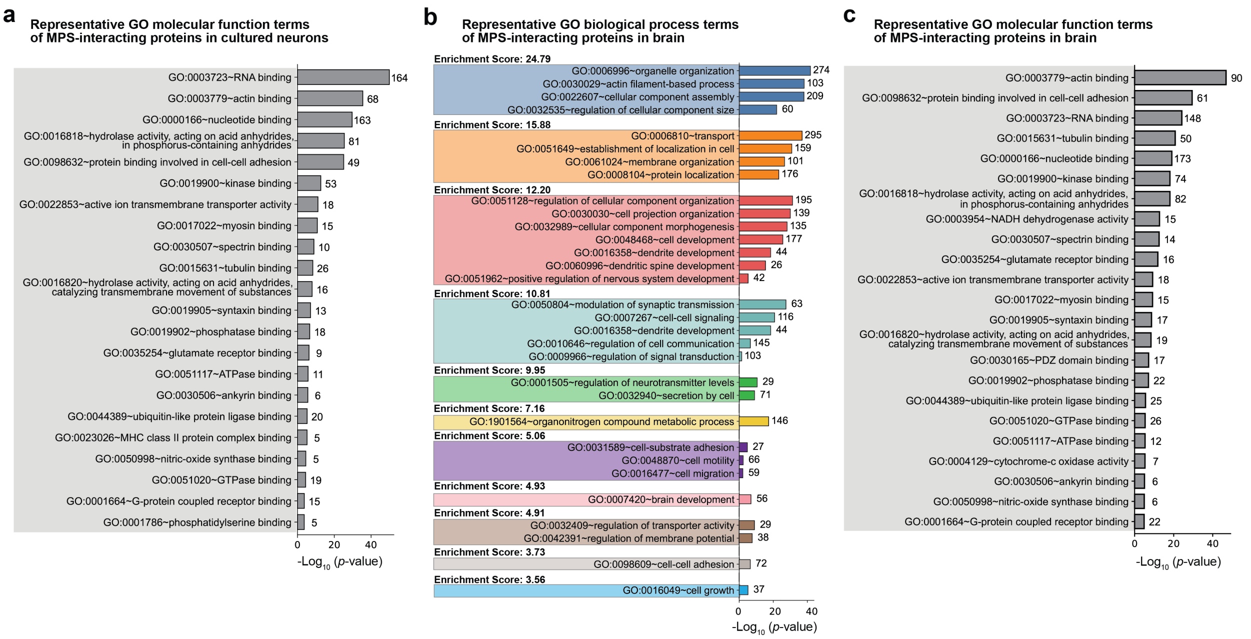

Supplementary Figure 2: GO term enrichment analyses of the candidate MPS-interacting proteins identified from cultured mouse hippocampal neurons and the adult mouse brain. a Enriched GO molecular function (MF) terms of the 480 candidate MPS-interacting proteins identified in cultured mouse hippocampal neurons. Enriched GO MF terms were generated using the DAVID 6.8 platform, and representative GO MF terms were shown with the corresponding *p*-values (bars) and the protein numbers (next to the bars) for each enriched GO MF term (See Supplementary Data 3 for the full list of enriched GO MF terms of the 480 candidate MPS-interacting proteins identified in cultured neurons). **b** Functional annotation clustering of the enriched GO terms in the biological process (BP) category for the 670 candidate MPS-interacting proteins identified in the adult mouse whole brain. Enriched GO BP terms were generated and clustered using the DAVID 6.8 platform with the enrichment score listed for each color-coded GO term cluster. A selected subset of GO BP terms for each GO term cluster were shown (See Supplementary Data 2 for the full list of clustered GO BP terms enriched in the 670 candidate MPS-interacting proteins identified in the adult mouse brain). The corresponding *p*-values (bars) and protein numbers (next to the bars) for each enriched GO BP term are shown on the right. **c** Same as (**a**) but for the 670 candidate MPS-interacting proteins identified in the adult mouse whole brain instead of in cultured neurons (See Supplementary Data 3 for the full list of enriched GO MF terms of the 670 candidate MPS-interacting proteins identified in the adult mouse brain).

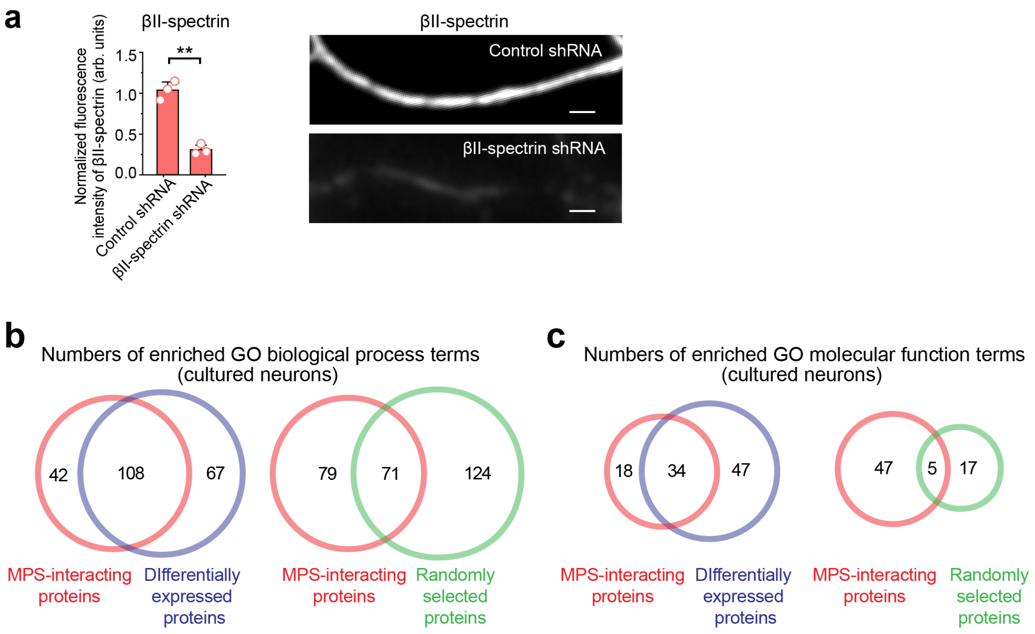

Supplementary Figure 3: Proteomic analyses for the differentially expressed proteins in MPS-disrupted neurons. a Average fluorescence intensities (left) and representative conventional images (right) of βII-spectrin in the axons of neurons transfected with adenoviruses expressing scrambled (control) shRNA and βII-spectrin shRNA. Data are mean ± s.e.m. (n = 3 biological replicates for each condition; 10-20 imaged regions were examined). ** indicates $p < 0.005$ (two-sided unpaired student's t-test); actual *p-value*: 2.1×10^{-3}. Images are representative examples from three independent experiments with similar results. Scale bars: 1 μm. **b** Left: Venn diagram showing the overlap between the enriched GO BP terms of the 480 candidate MPS-interacting proteins identified in cultured mouse hippocampal neurons (DIV 20) and the enriched GO BP terms of the 1,347 proteins differentially expressed in βII-spectrin knockdown neurons versus wildtype neurons (DIV 20). Right: Venn diagram showing the overlap between the enriched GO BP terms of the 480 candidate MPS-interacting proteins in cultured mouse hippocampal neurons and the enriched GO BP terms of 1347 randomly selected genes from the mouse genome (right). **c** Same as **b** but for GO MF terms instead of GO BP terms. See Supplementary Data 4 for the full list of differentially expressed proteins and Supplementary Data 5 and 6 for the corresponding enriched GO BP and MF terms, respectively. The 1347 differentially expressed proteins, with fold change >1.2 (for up regulation) or < 0.8 (for down regulation) in the βII-spectrin knockdown neurons as compared to the neurons treated with scrambled (control) shRNA, were determined using the DESeq2 package[1] to test the statistical significance for up and down regulation of each protein ($p < 0.01$ were used as the statistically significance threshold; n = 3 biological replicates for each condition). Source data are provided in the Source Data file.

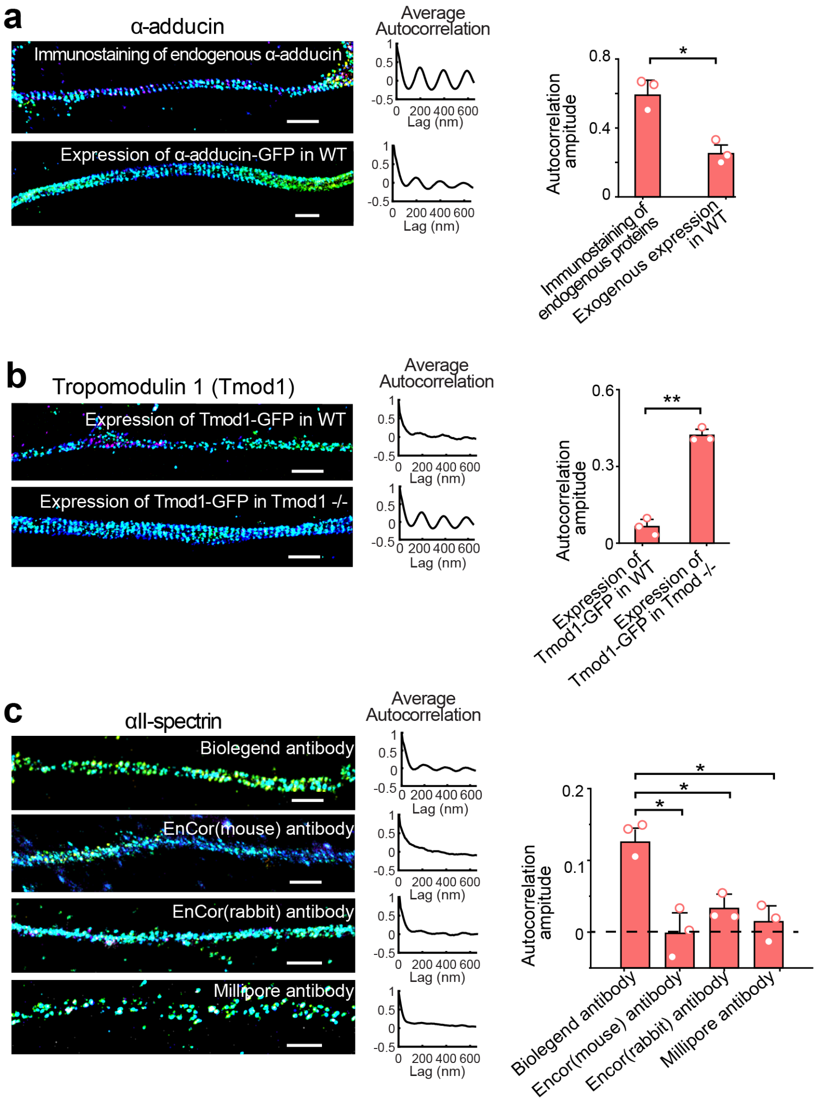

Supplementary Figure 4: Super-resolution imaging of MPS-interaction proteins using different labeling strategies. a Left: 3D STORM images of α-adducin in axons of cultured hippocampal neurons labeled using two different labeling strategies: 1) immunostaining with the antibody against a synthesized non-phosphopeptide derived from human α-adducin around the phosphorylation site of serine 726 (top); 2) labeled using moderate expression of GFP-tagged α-adducin, with GFP fused to the C-terminus of α-adducin, through low-titer lentiviral transfection, followed by immunolabeling using anti-GFP antibody (bottom). Middle: Average 1D autocorrelation of α-adducin over 20-50 randomly selected axon regions for the two strategies. Right: Average 1D auto-correlation amplitudes for the two strategies. * $p < 0.05$ (two-sided unpaired student's t-test); actual *p-value*: 7.6×10^{-3}. Data are mean ± s.e.m. (n = 3 biological replicates for each condition). Scale bars: 1 μm. **b** Same as (**a**) but for tropomodulin 1 labeled using the second strategy, with lentiviral transfection performed on either WT neurons (top) or tropomodulin 1

knockout neurons (bottom). GFP is fused to the C-terminus of tropomodulin 1. * $p < 0.05$ (two-sided unpaired student's t-test) and ** $p < 0.005$ (two-sided unpaired student's t-test); actual *p-value*: 1.8×10^{-4}. Data are mean ± s.e.m. ($n = 3$ biological replicates for each condition). **c** Same as (**a**) but for αII-spectrin labeled using the first strategy, with four different antibodies against αII-spectrin. The αII-spectrin antibodies obtained from Biolegend, Encor (mouse) and Encor (rabbit) are against the epitopes located at the SH3 domain, C-terminal 2 spectrin repeats, C-terminal 14 spectrin repeats of αII-spectrin, respectively, and the αII-spectrin antibody from Millipore is raised against chicken red blood cell membranes purified by hypotonic lysis and mechanical enucleation but the specific epitope location is unknown. * $p < 0.05$ (two-sided unpaired student's t-test); actual *p-value*s (from left to right): 1.3×10^{-2}, 2.1×10^{-2} and 5.8×10^{-3}. Data are mean ± s.e.m. ($n = 3$ biological replicates for each condition). Scale bars: 1 µm. STORM images in **a-c** are representative examples from three independent experiments with similar results. Source data are provided in the Source Data file.

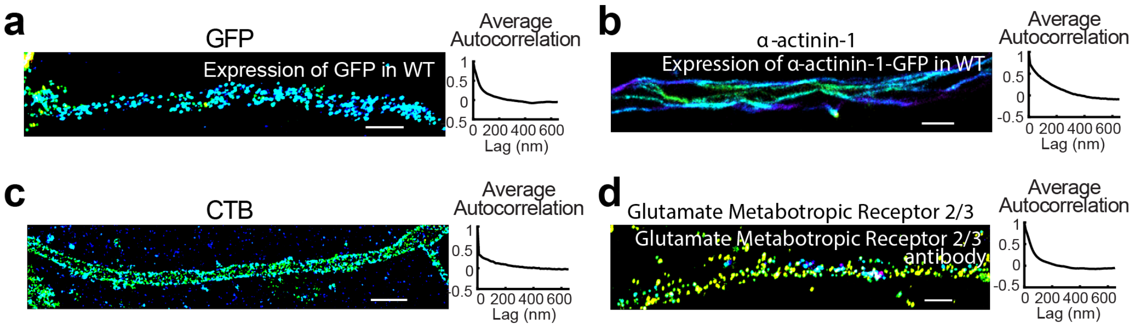

Supplementary Figure 5: Super-resolution imaging of control cytosolic and membrane proteins that do not exhibit periodic distributions. a Left: 3D STORM image of freely diffusing GFP molecules in the axon of cultured hippocampal neurons expressing GFP. GFP was immunolabeled using anti-GFP antibody. Average 1D autocorrelation function was calculated over 40-130 randomly selected axon regions. **b** Same as **a** but for α-actinin-1 instead of GFP. α-actinin-1 was labeled using moderate expression of GFP-tagged α-actinin-1 (with GFP fused to the C-terminus of α-actinin-1) through low-titer lentiviral transfection, followed by immunolabeling using anti-GFP antibody. **c** Same as **a** but for CTB-stained axon of cultured hippocampal neurons. CTB is known to bind to ganglioside GM1 enriched in the plasma membrane. **d** Same as **a** but for glutamate metabotropic receptor 2/3 instead of GFP. Glutamate metabotropic receptor 2/3 was immunostained with antibody raised against the C-terminus of rat glutamate metabotropic receptor 2. STORM images are representative examples from three independent experiments with similar results. Scale bars: 1 µm. Source data are provided in the Source Data file.

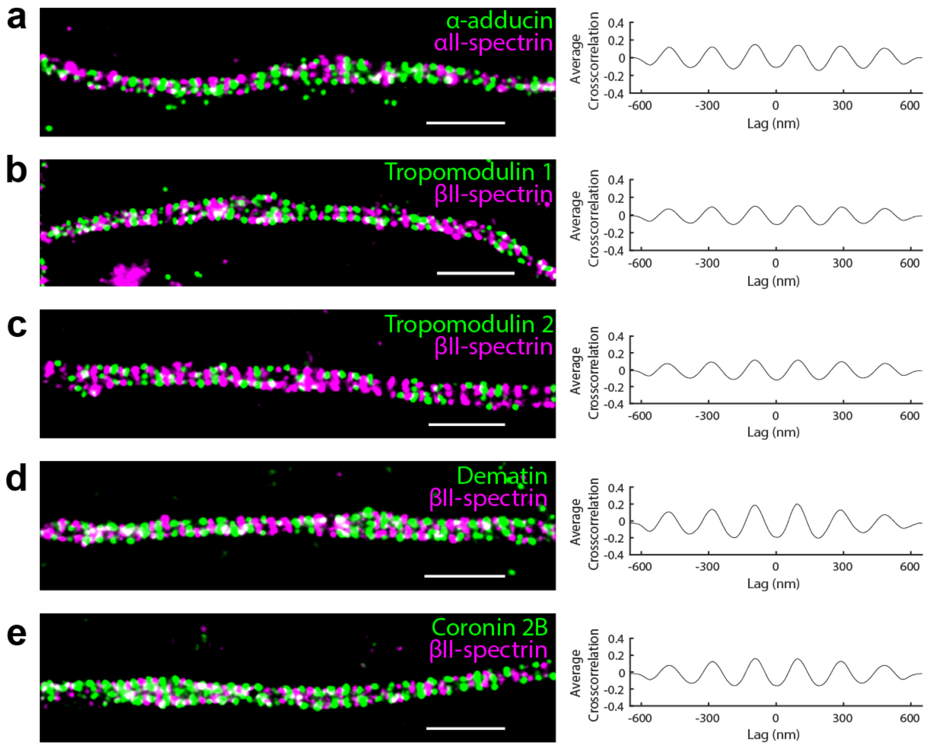

Supplementary Figure 6: Spatial relationship between several MPS structural components. a Left: Two-color STORM images of αII-spectrin (magenta) and α-adducin (green) in the axons of cultured hippocampal neurons. Scale bar: 1 μm. Right: Average 1D cross-correlation function between the distributions of α-adducin and αII-spectrin (40-100 axonal regions were analyzed). **b** Similar to (**a**) but for βII-spectrin (magenta) and tropomodulin 1 (green). **c** Similar to (**a**) but for βII-spectrin (magenta) and tropomodulin 2 (green). **d** Similar to (**a**) but for βII-spectrin (magenta) and dematin (green). **e** Similar to (**a**) but for βII-spectrin (magenta) and coronin 2B (green). αII-spectrin, βII-spectrin, α-adducin, dematin, and coronin 2B were immunolabeled with their corresponding antibodies against the SH3 domain of human αII-spectrin, C-terminal amino acids 2101-2189 of human βII-spectrin, and the C-terminal amino acids 724-728 of human α-adducin, the N-terminal amino acids 68-190 of human dematin, and the C-terminal amino acids 364-467 of human coronin 2B, respectively. Tropomodulin 1 and tropomodulin 2 were labeled using moderate expression of GFP-tagged proteins, with GFP fused to the C-terminus of the target protein, through low-titer lentiviral transfection. The GFP was in turn immunolabeled using anti-GFP antibody. STORM images in **a-e** are representative examples from three independent experiments with similar results. Source data are provided in the Source Data file.

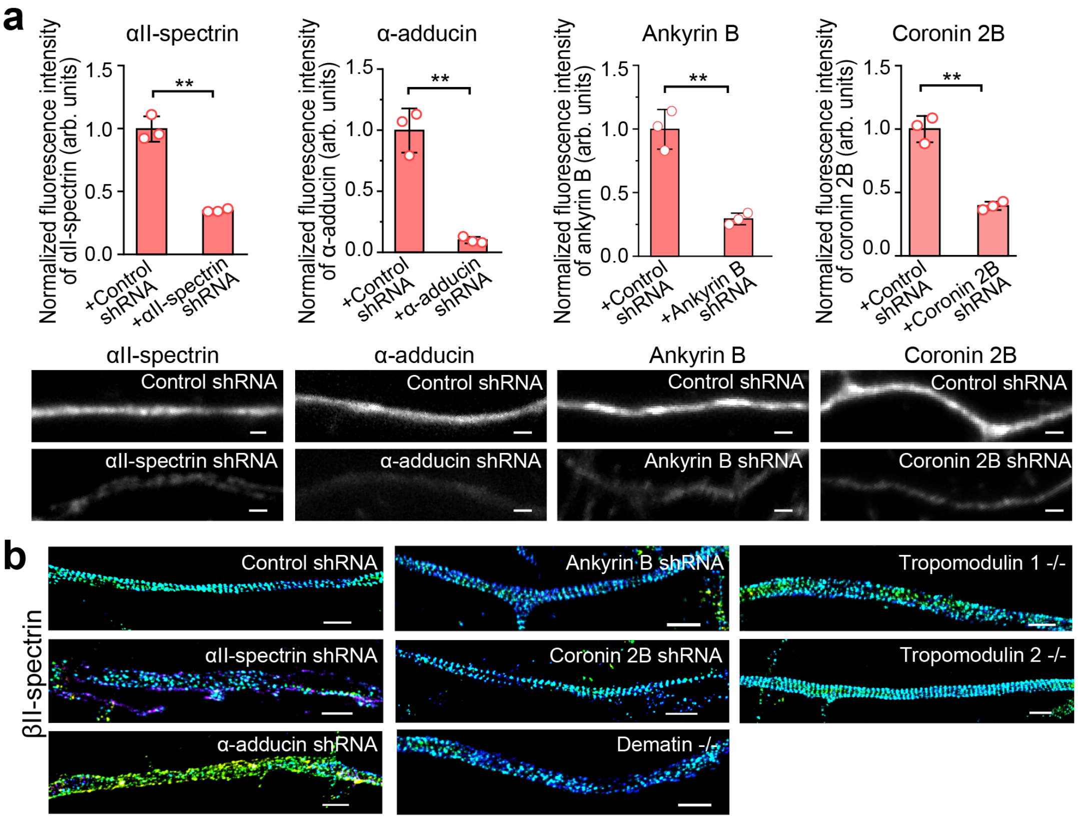

Supplementary Figure 7: Determination of proteins essential for MPS formation and maintenance by gene knockdown and knockout. a Average fluorescence intensities (top) and representative conventional images (bottom) of αII-spectrin, α-adducin, ankyrin B, and coronin 2B in the axons of neurons transfected with adenoviruses expressing scrambled (control) shRNA or the shRNA against the corresponding protein, showing the knockdown of αII-spectrin, α-adducin, ankyrin B, and coronin 2B. Data are mean ± s.e.m. m (n = 3 biological replicates; 10-20 imaged regions were examined per condition). *p < 0.05, **p < 0.005 (two-sided unpaired student's t-test); actual *p-value*s (from left to right): 4.7×10^{-3}, 1.2×10^{-3}, 1.7×10^{-4} and 1.3×10^{-3}. Scale bars: 1 μm. **b** 3D STORM images of βII-spectrin in axon segments of cultured hippocampal neurons treated with control shRNA, αII-spectrin shRNA, α-adducin shRNA, ankyrin B shRNA, coronin 2B shRNA, and of neurons cultured from dematin, tropomodulin 1, and tropomodulin 2 knockout mice. Quantification of the autocorrelation amplitudes of the βII-spectrin distributions, reflecting the degree of periodicity of the MPS under these conditions, is shown in Figure 2f. Images are representative examples from three independent experiments with similar results. Scale bars: 1 μm. Source data are provided in the Source Data file.

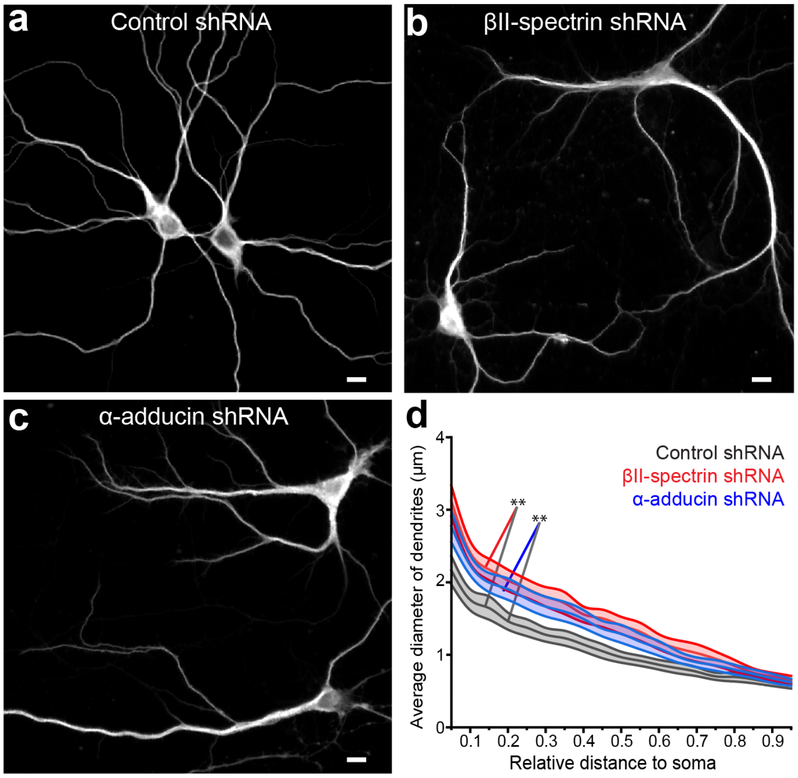

Supplementary Figure 8: Effect of the MPS disruption on the diameters of dendrites. a-c Representative conventional images of cultured hippocampal neurons treated with control shRNA(**a**), βII-spectrin shRNA(**b**), and α-adducin shRNA (**c**). The neurons were immunolabeled with the dendritic marker MAP2. Scale bars: 10 μm. **d** Average diameters of dendrites as a function of the relative distance to the soma for the conditions described in (**a-c**). Shaded regions represent s.e.m. from the mean for each condition ($n = 3$ biological replicates; 20-30 images were analyzed). ** indicates $p < 0.005$ (two-sided paired student's t-test); actual *p-value*s (from left to right): 2.5×10^{-7} and 5.0×10^{-7}. Images in **a-c** are representative examples from three independent experiments with similar results. Source data are provided in the Source Data file.

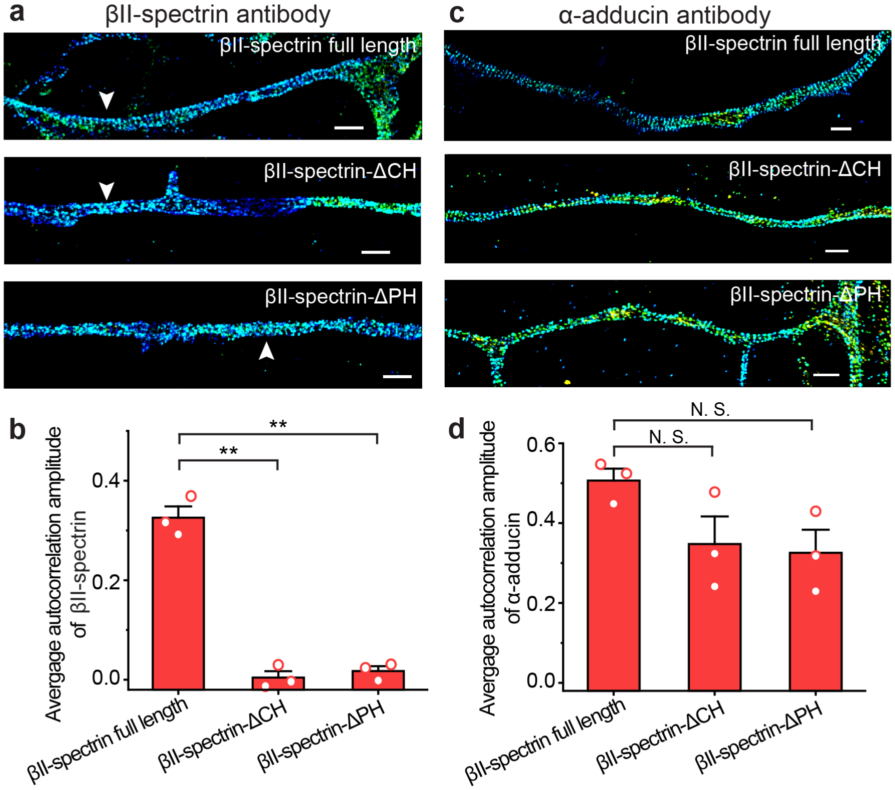

Supplementary Figure 9: Role of the CH and PH domains of βII-spectrin in MPS formation. a 3D STORM image of βII-spectrin or βII-spectrin mutants in the axons of wild-type neurons transfected with plasmid expressing GFP-tagged full length βII-spectrin (top), βII-spectrin-ΔCH mutant (middle) or βII-spectrin-ΔPH mutant (bottom). The proteins were visualized by immunostained with anti-GFP antibody. Scale bars: 1 µm. **b** Average 1D auto-correlation amplitudes for the distribution of GFP-tagged full length βII-spectrin and the distributions of the two GFP-tagged βII-spectrin truncation mutants, as described in (**a**). Data are mean ± s.e.m. (n = 3 biological replicates for each condition; 10-30 axonal regions were examined for each condition). ** indicates $p < 0.005$ (two-sided unpaired Student's t-test); actual *p-value*s (from left to right): 3.3×10^{-3} and 1.5×10^{-3}. **c, d** Similar to (**a, b**) but the axons were immunostained with anti-α-adducin antibody instead of anti-GFP antibody. The overexpression of the truncated βII-spectrin variants in wildtype neurons did not significantly affect the endogenous MPS structures, as probed by the anti-α-adducin antibody. N.S. indicates $p > 0.05$ (two-sided unpaired Student's t-test); actual *p-value*s (from left to right): 0.12 and 0.09. STORM images in **a** and **c** are representative examples from three independent experiments with similar results. Source data are provided in the Source Data file.

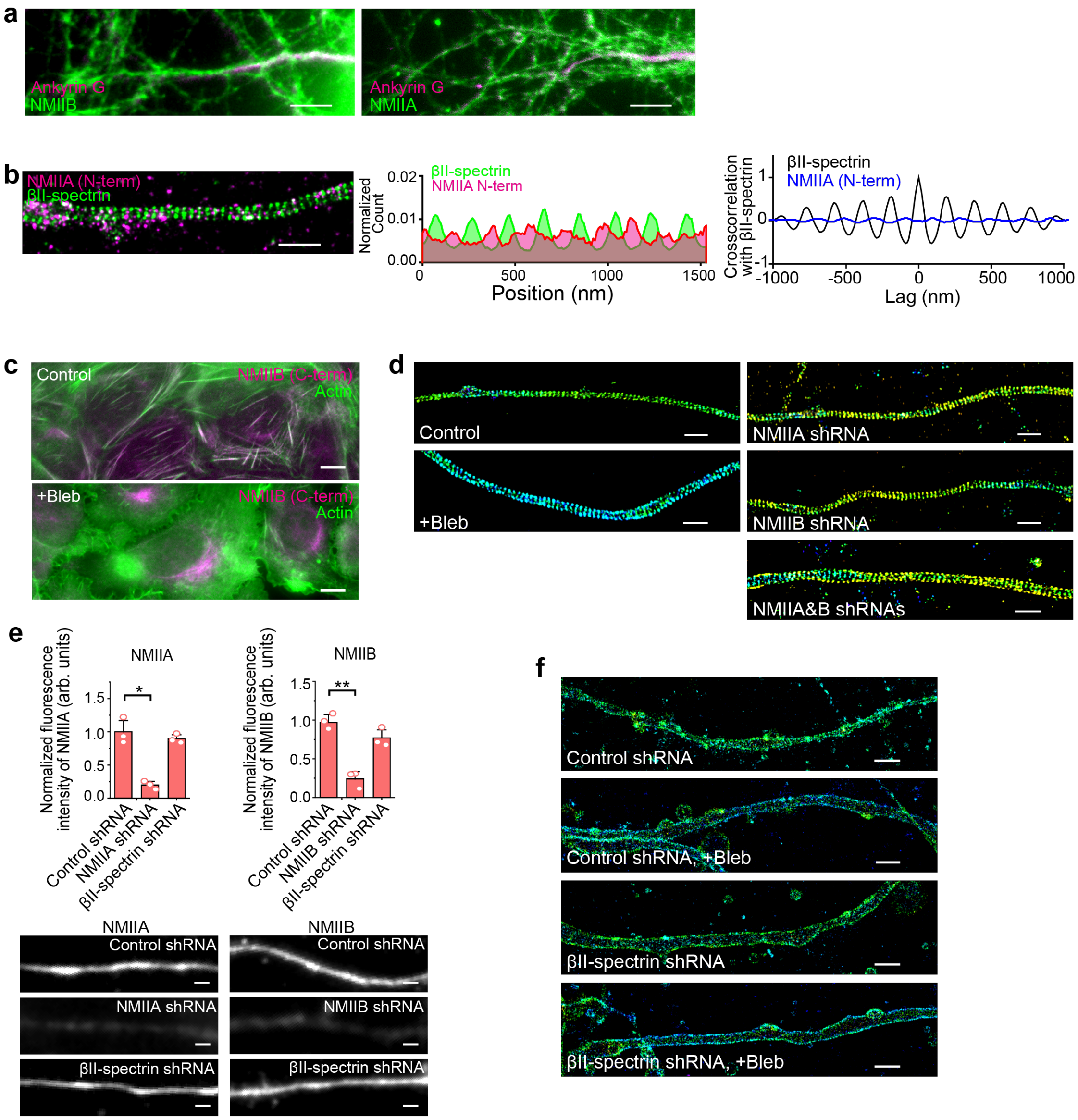

Supplementary Figure 10: Spatial distribution of NMIIB and NMIIA in axons and effects of NMII activity inhibition on the MPS and axon diameters. **a** Left: Conventional fluorescence image of NMIIB (green, left) or NMIIA (green, right) and the AIS marker ankyrin G (magenta) in axons of cultured neurons. Scale bars: 10 µm. **b** Left: Two-color STORM image of βII-spectrin (C-terminus, green) and NMIIA (N-terminus, magenta) in the axons. Middle: Average 1D distribution of βII-spectrin (C-terminus, green) and NMIIA (N-terminus, magenta) signals projected to the longitudinal axon axis, derived from 34 axon regions. Right: Average 1D cross-correlation between the distributions of βII-spectrin (C-terminus) and NMIIA (N-terminus) (blue). Average 1D autocorrelation of βII-spectrin distribution (black) is reproduced from Fig. 3d as a reference. Scale bars: 1 µm. **c** Conventional fluorescence images of actin (green) and NMIIB (C-terminus, magenta) in untreated U2OS cells (top) and U2OS cells treated with blebbistatin (Bleb) for 2

hours (bottom). Actin was stained with phalloidin. Scale bars: 10 μm. **d** 3D STORM images of βII-spectrin in the axons of control untreated neurons, neurons treated with Bleb, and neurons treated with NMIIA heavy chain shRNA, NMIIB heavy chain shRNA or both shRNAs. Scale bars: 1 μm. **e** Average fluorescence intensities (top) and representative images (bottom) of NMIIA and NMIIB in the axons of neurons transfected with adenoviruses expressing shRNA against NMIIA heavy chain, NMIIB heavy chain, or βII-spectrin, in comparison with neurons transfected with adenoviruses expressing scrambled (control) shRNA. Data are mean ± s.e.m. ($n = 3$ biological replicates for each condition; 10-20 imaged regions were examined). ** $p < 0.005$ (two-sided unpaired student's t-test); actual *p-value*s (from left to right): 1.3×10^{-2} and 1.4×10^{-3}. **f** 3D STORM images of the cholera toxin B (CTB)-stained axons of neurons treated with control (scramble) shRNA, neurons treated with control shRNA and Bleb, neurons treated with βII-spectrin shRNA, and neurons treated with βII-spectrin shRNA and Bleb. Scale bars: 1 μm. The conventional fluorescence and STORM images are representative examples of three independent experiments with similar results. Source data are provided in the Source Data file.

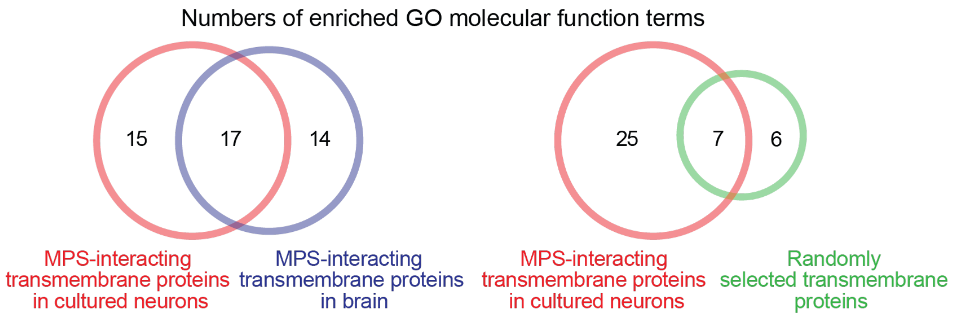

Supplementary Figure 11: Comparison of the enriched GO terms for the candidate MPS-interacting transmembrane proteins identified in the cultured mouse hippocampal neurons and those identified in the mouse whole brain. Venn diagrams showing the overlap between the enriched GO MF terms for the 95 candidate MPS-interacting transmembrane proteins identified in cultured mouse hippocampal neurons (DIV 20) and the enriched GO MF terms for the 176 candidate MPS-interacting transmembrane proteins identified in the adult mouse whole brain (left). Also show in comparison is the overlap between the enriched GO MF terms for the 95 candidate MPS-interacting transmembrane proteins identified in cultured mouse hippocampal neurons (DIV 20) and the enriched GO MF terms for 176 randomly selected transmembrane proteins from the mouse genome (right).

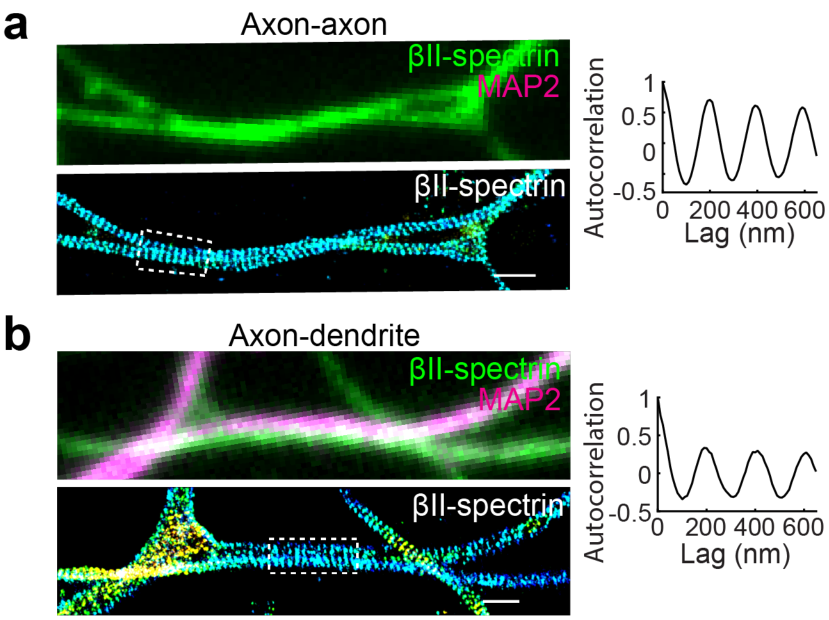

Supplementary Figure 12: MPS alignment between abutting neurites. a-b Top left: conventional fluorescence images of cultured neurons immunostained for βII-spectrin (green) and MAP2 (magenta). Bottom left: 3D STORM images of βII-spectrin in the same region as shown in the top left panel, exhibiting MPS alignment at the axon-axon (**a**) and axon-dendrite (**b**) contact sites, exemplified by regions in the dashed boxes. Right: 1D autocorrelation function of the boxed regions in the bottom left panels. Images are representative examples from three independent experiments with similar results. Scale bars: 1 μm. Source data are provided in the Source Data file.

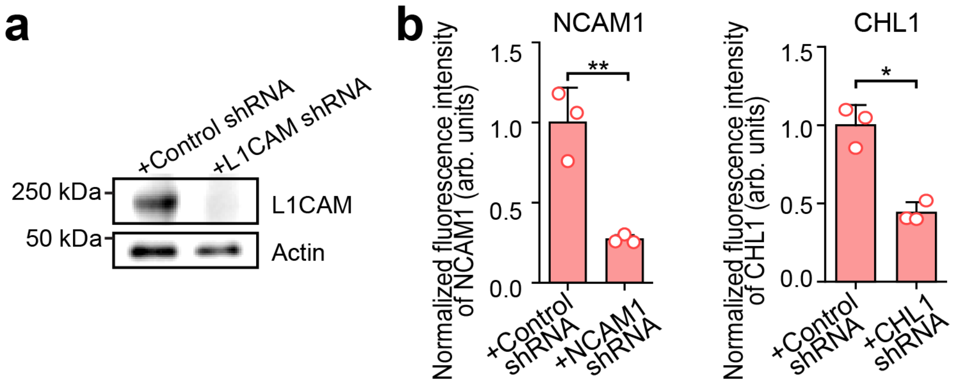

Supplementary Figure 13: Knockdown of cell adhesion molecules. **a** Western blot showing the knockdown of L1CAM in the cultured neurons transfected with adenoviruses expressing L1CAM shRNA in comparison with neurons transfected with adenoviruses expressing control shRNA. The Western blot is a representative example from two independent experiments with similar results. **b** Average fluorescence intensities of NCAM1 and CHL1 in the axons of neurons transfected with adenoviruses expressing scrambled (control) shRNA or the shRNA against the corresponding protein (NCAM1 and CHL1). Data are mean ± s.e.m. ($n = 3$ biological replicates; 10-20 imaged regions were examined per condition). * $p < 0.05$, ** $p < 0.005$ (two-sided unpaired student's t-test); actual *p-value*s (from left to right): 6.9×10^{-3} and 2.6×10^{-2}. Source data are provided in the Source Data file.

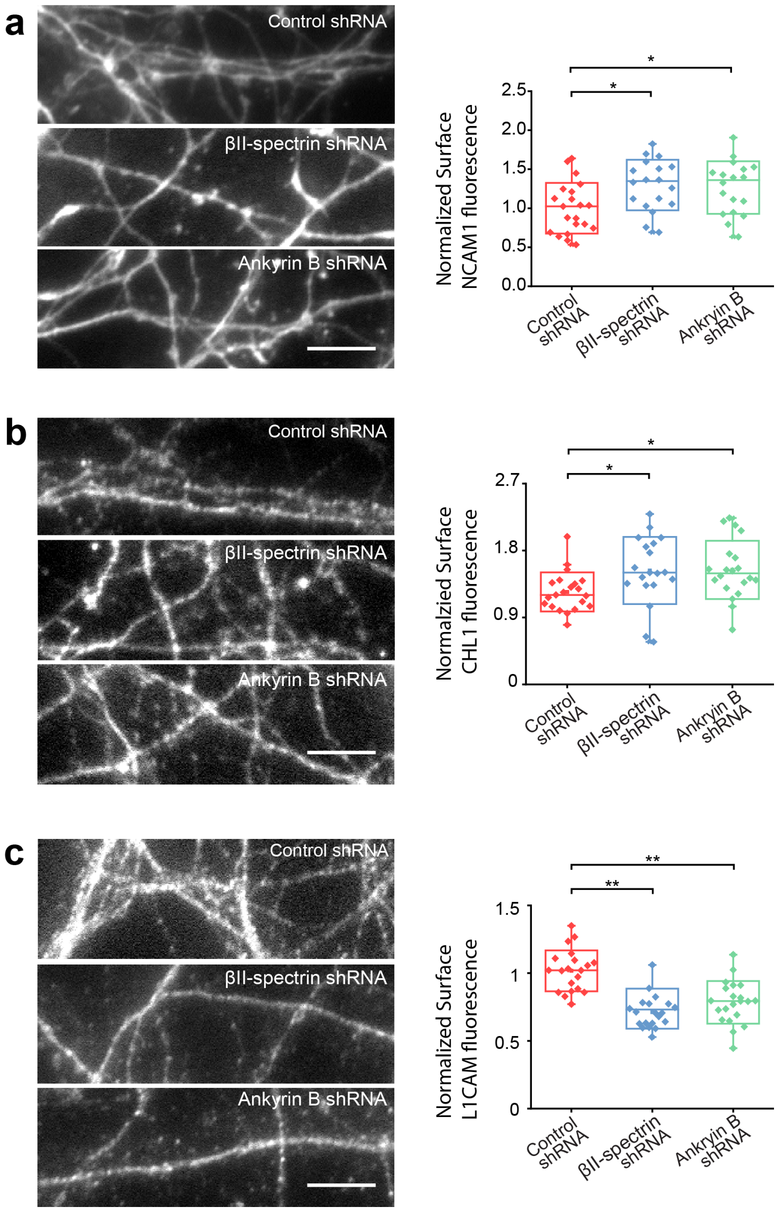

Supplementary Figure 14: Cell-surface expression levels of NCAM1, CHL1 and L1CAM in control, βII-spectrin knockdown and ankyrin B knockdown neurons. a Left: Fluorescence images of cell-surface NCAM1 for the neurons transfected with adenoviruses expressing scrambled (control) shRNA (top panel), βII-spectrin shRNA (middle panel) and ankyrin B shRNA (bottom panel). Right: Box plots of the cell-surface NCAM1 expression levels at the neurites for neurons transfected with adenoviruses expressing control shRNA (red), βII-spectrin shRNA (blue) and ankyrin B shRNA (green). * $p < 0.05$ and ** $p < 0.005$ (two-sided unpaired student's t-test); actual p values (from left to right): 1.0×10^{-2} and 1.7×10^{-2}. ($n = 3$ biological replicates; 15-25 imaged regions were examined). The line in the middle of the box indicates the median value; the lower and upper bounds of the box indicate 25th and 75th percentile; the whiskers indicate

the minimum and maximum. Scale bars: 10 µm. **b** Same as (**a**) but for the cell-surface expression levels of CHL1 instead of NCAM1. * $p < 0.05$ and ** $p < 0.005$ (two-sided unpaired student's t-test); actual *p-value*s (from left to right): 2.1×10^{-2} and 0.9×10^{-3}. **c** Same as (**a**) but for the cell-surface expression levels of L1CAM instead of NCAM1. ** $p < 0.005$ (two-sided unpaired student's t-test); actual *p-value*s (from left to right): 1.5×10^{-4} and 4.9×10^{-3}. Images are representative examples from three independent experiments with similar results. Source data are provided in the Source Data file.

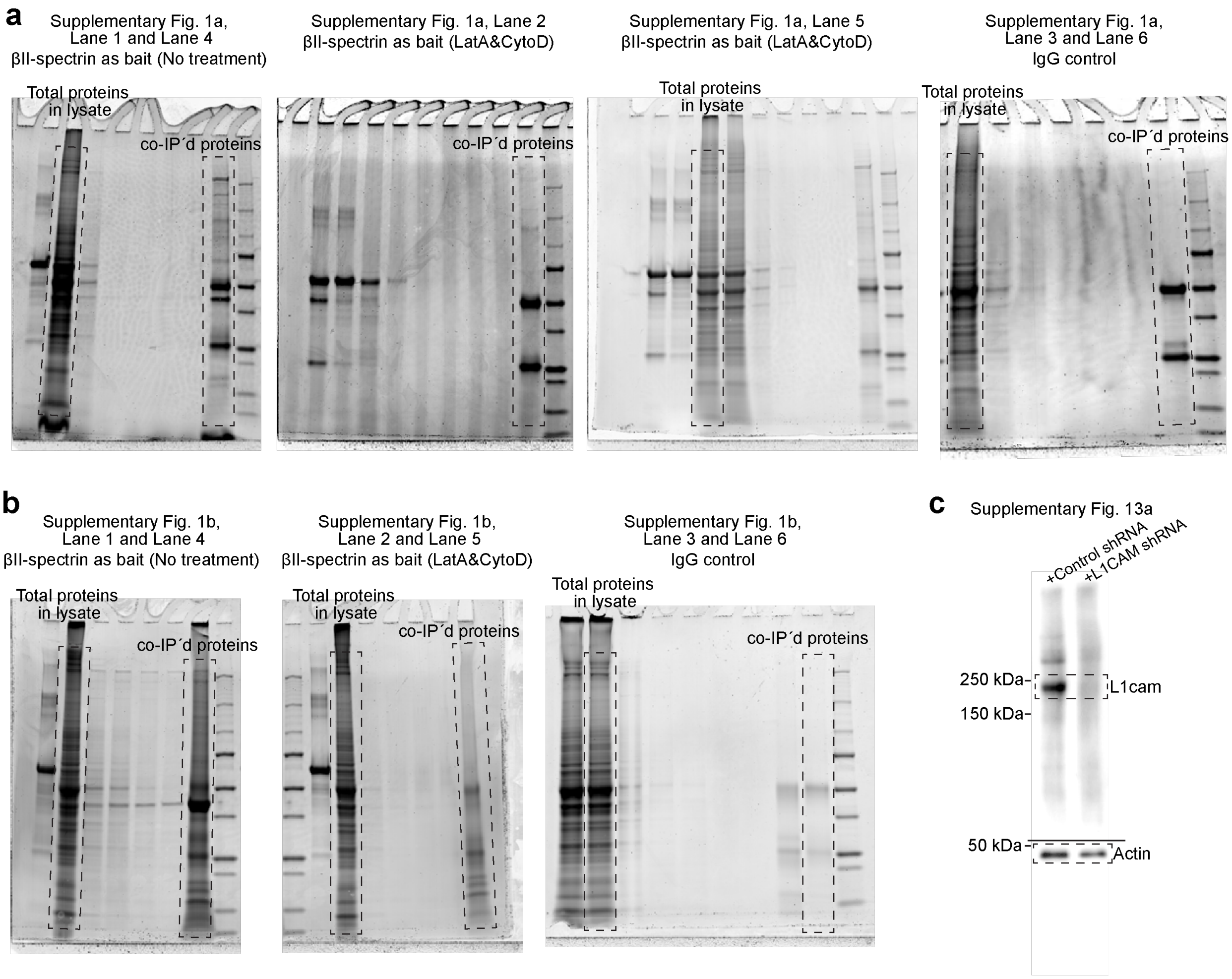

Supplementary Figure 15: Uncropped SDS-PAGE and Western blot images for supplementary figures. a Uncropped SYPRO Red-stained SDS-PAGE images for Supplementary Fig. 1a. Dashed boxes indicate the cropped regions in Supplementary Fig. 1a. **b** Uncropped SYPRO Red-stained SDS-PAGE images for Supplementary Fig. 1b. Dashed boxes indicate the cropped regions shown in Supplementary Fig. 1b. **c** Uncropped Western blot image for Supplementary Fig. 13a, showing the knockdown of L1CAM in the cultured neurons transfected with adenoviruses expressing L1CAM shRNA in comparison with neurons transfected with adenoviruses expressing control shRNA. Solid black line indicates the position where the membrane was cut into two pieces (upper piece for L1CAM immunostaining and lower piece for actin immunostaining) before incubation with antibody. Dashed boxes indicate the cropped regions shown in Supplementary Fig. 13a.

Supplementary Reference

1. Love, M.I., Huber, W. & Anders, S. Moderated estimation of fold change and dispersion for RNA-seq data with DESeq2. *Genome Biol* **15**, 550 (2014).
