# Supplementary material for: Proteomic and functional analyses of the periodic membrane skeleton in neurons
Source: Nat Commun. 2022 Jun 9;13:3196. doi: 10.1038/s41467-022-30720-x (PMC9184744; doi:10.1038/s41467-022-30720-x)
Supplement: Supplementary file 3 — Description of Additional Supplementary Files [file 41467_2022_30720_MOESM3_ESM.pdf]

Description of Additional Supplementary Files

File Name: Supplementary Data 1

Description: Lists of the potential candidate MPS-interacting proteins identified by co-IP and mass spectrometry analysis. Candidate MPS-interacting proteins identified from the capture co-IP experiments using αII-spectrin, βII-spectrin, or α-adducin as the bait are shown in three separated spreadsheets (Cultured neuron capture co-IP, Brain capture co-IP, and Overlap (culture neuron&brain)): the first spreadsheet contains the protein list identified from cultured mouse hippocampal neurons (DIV 20), the second spreadsheet contains the protein list identified from adult mouse whole-brain, and the third spreadsheet contains the overlapped proteins in the first and second spreadsheets. Only the candidate proteins that were commonly identified using all three baits are listed. The proteins identified in the negative control co-IP experiments using the irrelevant (control) IgG antibody are shown in two separate spreadsheets (Cultured neuron control co-IP and Brain control co-IP): one for the proteins identified in the negative control co-IP from cultured mouse hippocampal neurons and the other for the proteins identified in the negative control co-IP from adult mouse whole-brain, and only the proteins commonly detected in all capture co-IP experiments using the three bait proteins and the control co-IP experiments are listed. The Q-values (false discovery rate), Sum PEP scores (the protein score calculated as the sum of the negative logarithms of the posterior error probability (PEP: the probability that the observed peptide spectral count is incorrect) values of the connected peptide spectrum matches), and Score Sequest HT values (The sum of the scores of the individual peptides obtained from the Sequest HT search) are provided for all the identified proteins. The calculated dNSAF values are shown as a label free quantitative measure of protein abundance. In the spreadsheet for the negative control co-IP experiments using IgG, we also provide the calculated fold-changes of dNSAF values between the capture co-IP experiment with the proper bait protein and the negative control co-IP experiment using the IgG. Proteins with the dNSAF fold-change values that were not greater than 1 were considered non-specifically pulled down and were not included in the first three lists (Cultured neuron capture co-IP, Brain capture co-IP, and Overlap (culture neuron&brain)).

File Name: Supplementary Data 2

Description: Enriched GO biological process (BP) terms of the candidate MPS-interacting proteins. Enriched GO BP terms were generated and clustered using the DAVID 6.8 platform with the enrichment score listed for each GO term cluster. The first spreadsheet (Cultured neurons) contains the GO BP terms generated for the candidate MPS-interacting proteins list identified from cultured mouse hippocampal neurons (DIV 20), and the second spreadsheet (Brain) contains the protein list identified from adult mouse whole-brain. The unclustered GO BP terms are listed in the end of each spreadsheet. For each GO BP term, the involved gene count (i.e., the number of the protein hits), the percentage of the involved genes over

total genes in this GO term as well as the involved gene names are listed, along with the one-sided Fisher-exact *p*-values and fold-enrichment values to measure the significance and the magnitude of the enrichment, respectively. The representative GO BP terms in each GO term cluster shown in Fig. 1c and Supplementary Fig. 2b are highlighted in yellow.

File Name: Supplementary Data 3

Description: Enriched GO molecular function (MF) terms of the candidate MPS-interacting proteins. Enriched GO MF terms were generated using the DAVID 6.8 platform. The first spreadsheet (Cultured neurons) contains the GO MF terms generated for the candidate MPS-interacting proteins list identified from cultured mouse hippocampal neurons (DIV 20), and the second spreadsheet (Brain) contains the protein list identified from adult mouse whole-brain. For each GO MF term, the involved gene count (i.e., the number of the protein hits), the percentage of the involved genes over total genes in this GO term as well as the involved gene names are listed, along with the one-sided Fisher-exact *p*-value and fold-enrichment value to measure the statistical significance and the magnitude of the enrichment, respectively. The representative GO MF terms in each GO term cluster shown in Supplementary Fig. 2a,c are highlighted in yellow.

File Name: Supplementary Data 4

Description: List of the differentially expressed proteins in βII-spectrin knockdown neurons identified by quantitative mass spectrometry and DESeq2 analyses. The abundances of the differentially expressed gene under the two conditions (cultured neurons treated with scrambled (control) shRNA or βII-spectrin shRNA) measured by quantitative mass spectrometry are listed. For each gene, the fold change of the abundance and the *p*-value (two-sided Wald test) are listed along with other output parameters from the DESeq2 package (Version 1.34.0) measuring the statistical significance. The differentially expressed proteins are defined with abundance fold change >1.2 (for up regulation) or < 0.8 (for down regulation) in the βII-spectrin knockdown neurons as compared to the neurons treated with scrambled (control) shRNA ($p < 0.01$).

File Name: Supplementary Data 5

Description: Enriched GO BP terms of the differentially expressed genes in βII-spectrin knockdown neurons. Enriched GO BP terms were generated and clustered using the DAVID 6.8 platform with the enrichment score listed for each GO term cluster. For each GO BP term, the involved gene count (i.e., the number of the protein hits), the percentage of the involved genes over total genes in this GO term as well as the involved gene names are listed, along with the one-sided Fisher-exact *p*-value and fold-enrichment value to measure the significance and the magnitude of the enrichment, respectively.

File Name: Supplementary Data 6

Description: Enriched GO MF terms of the differentially expressed genes in β II-spectrin knockdown neurons. Enriched GO MF terms were generated using the DAVID 6.8 platform. For each GO MF term, the involved gene count (i.e., the number of the protein hits), the percentage of the involved genes over total genes in this GO term as well as the involved gene names are listed, along with the one-sided Fisher-exact *p*-values and fold-enrichment values to measure the statistical significance and the magnitude of the enrichment, respectively.

File Name: Supplementary Data 7

Description: List of candidate MPS-interacting transmembrane proteins identified by co-IP and mass spectrometry analysis. Candidate MPS-interacting transmembrane proteins identified from the co-IP experiments using αII-spectrin, βII-spectrin, or α-adducin as the bait are shown in two separated spreadsheets (Cultured neuron capture co-IP and Brain capture co-IP): the first spreadsheet contains the protein list identified from cultured mouse hippocampal neurons (DIV 20), the second spreadsheet contains the protein list identified from adult mouse whole-brain. The candidate transmembrane proteins that were identified using at least one of the three baits are listed. The Q-values, Sum PEP scores, and Score Sequest HT values are provided for all the identified proteins. The calculated dNSAF values are shown as a label free quantitative measure of protein abundance. For each co-IP experiment (i.e., αII-spectrin, βII-spectrin, or α-adducin capture experiment), we calculated the fold-changes of dNSAF values between the capture co-IP experiment using the proper bait protein and the negative control co-IP experiment using IgG. Proteins with the dNSAF fold-change values that were not greater than 1 were considered non-specifically pulled down and were not included in these lists.

File Name: Supplementary Data 8

Description: Enriched GO MF terms of the candidate MPS-interacting transmembrane proteins. Enriched GO MF terms were generated using the DAVID 6.8 platform. The first spreadsheet (MF GO terms of cultured neurons) contains the GO MF terms generated for the candidate MPS-interacting proteins list identified from cultured mouse hippocampal neurons (DIV 20), and the second spreadsheet (MF GO terms of brain) contains the GO MF terms generated for the protein list identified from adult mouse whole-brain. For each GO MF term, the involved gene count (i.e., the number of the protein hits), the percentage of the involved genes over total genes in this GO term as well as the involved gene names are listed, along with the one-sided Fisher-exact *p*-values and fold-enrichment values to measure the statistical significance and the magnitude of the enrichment, respectively.
