# Supplementary material for: Proteomic and functional analyses of the periodic membrane skeleton in neurons
Source: Nat Commun. 2022 Jun 9;13:3196. doi: 10.1038/s41467-022-30720-x (PMC9184744; doi:10.1038/s41467-022-30720-x)
Supplement: Supplementary file 12 — Reporting Summary [file 41467_2022_30720_MOESM12_ESM.pdf]

nature portfolio

Corresponding author(s): Xiaowei Zhuang & Ruobo Zhou

Last updated by author(s): May 1, 2022

Reporting Summary

Nature Portfolio wishes to improve the reproducibility of the work that we publish. This form provides structure for consistency and transparency in reporting. For further information on Nature Portfolio policies, see our Editorial Policies and the Editorial Policy Checklist.

Statistics

For all statistical analyses, confirm that the following items are present in the figure legend, table legend, main text, or Methods section.

n/a	Confirmed	
☐	☒	The exact sample size (n) for each experimental group/condition, given as a discrete number and unit of measurement
☐	☒	A statement on whether measurements were taken from distinct samples or whether the same sample was measured repeatedly
☐	☒	The statistical test(s) used AND whether they are one- or two-sided<br>*Only common tests should be described solely by name; describe more complex techniques in the Methods section.*
☒	☐	A description of all covariates tested
☒	☐	A description of any assumptions or corrections, such as tests of normality and adjustment for multiple comparisons
☐	☒	A full description of the statistical parameters including central tendency (e.g. means) or other basic estimates (e.g. regression coefficient) AND variation (e.g. standard deviation) or associated estimates of uncertainty (e.g. confidence intervals)
☐	☒	For null hypothesis testing, the test statistic (e.g. F, t, r) with confidence intervals, effect sizes, degrees of freedom and P value noted<br>*Give P values as exact values whenever suitable.*
☒	☐	For Bayesian analysis, information on the choice of priors and Markov chain Monte Carlo settings
☒	☐	For hierarchical and complex designs, identification of the appropriate level for tests and full reporting of outcomes
☒	☐	Estimates of effect sizes (e.g. Cohen's d, Pearson's r), indicating how they were calculated

Our web collection on statistics for biologists contains articles on many of the points above.

Software and code

Policy information about availability of computer code

Data collection	Software used for STORM data collection is available at https://github.com/ZhuangLab/storm-control and Zenodo (DOI: 10.5281/zenodo.3264857).
Data analysis	Data analysis code are available at https://github.com/boranhan/MPS and Zenodo (DOI: 10.5281/zenodo.6513278). Additional Software used: Matlab (Version R2018b), ImageJ (Version 1.53) , DESeq2 (Version 1.34.0), Insight3 (a previously reported algorithm), DAVID (Version 6.8, https://david.ncifcrf.gov/), Proteome Discoverer (Version 2.4).

For manuscripts utilizing custom algorithms or software that are central to the research but not yet described in published literature, software must be made available to editors and reviewers. We strongly encourage code deposition in a community repository (e.g. GitHub). See the Nature Portfolio guidelines for submitting code & software for further information.

Data

Policy information about availability of data

All manuscripts must include a data availability statement. This statement should provide the following information, where applicable:

- Accession codes, unique identifiers, or web links for publicly available datasets
- A description of any restrictions on data availability
- For clinical datasets or third party data, please ensure that the statement adheres to our policy

All data supporting the findings of this study are provided within the paper and its supplementary information. The fasta file of the mouse proteome (Uniprot Mus musculus proteome UP000000589) was downloaded from Uniprot (https://www.uniprot.org/proteomes/UP000000589). The mass spectrometry proteomics data have been deposited to the ProteomeXchange Consortium via the PRIDE partner repository with the data set identifier PXD030886 (https://www.ebi.ac.uk/pride/archive/projects/PXD030886). Source data of all data presented in graphs within the figures are provided with this paper. Uncropped gel images are shown in

Supplementary Figure 15.

Field-specific reporting

Please select the one below that is the best fit for your research. If you are not sure, read the appropriate sections before making your selection.

[x] Life sciences   [ ] Behavioural & social sciences   [ ] Ecological, evolutionary & environmental sciences

For a reference copy of the document with all sections, see nature.com/documents/nr-reporting-summary-flat.pdf

Life sciences study design

All studies must disclose on these points even when the disclosure is negative.

Sample size	Sample sizes in imaging experiments were determined based on standards for the imaging-based studies in the field of cell biology, and provide sufficient statistics.
Data exclusions	No data from samples were excluded from analyses.
Replication	Three biological replicates were acquired for each condition of the imaging experiments. For co-IP based mass-spec analysis, two biological replicates were acquired for each condition, and for the quantitative TMT-based mass-spec analysis, three biological replicates were acquired for each condition.
Randomization	The study does not require the allocation of samples into different experimental groups. Hence, randomization was not needed or performed for this study.
Blinding	Blinding was not done because the imaging samples were prepared and analyzed by the same investigator due to the sophistication required in each experiment.

Reporting for specific materials, systems and methods

We require information from authors about some types of materials, experimental systems and methods used in many studies. Here, indicate whether each material, system or method listed is relevant to your study. If you are not sure if a list item applies to your research, read the appropriate section before selecting a response.

Materials & experimental systems

n/a	Involved in the study	
[ ]	[x]	Antibodies
[ ]	[x]	Eukaryotic cell lines
[x]	[ ]	Palaeontology and archaeology
[ ]	[x]	Animals and other organisms
[x]	[ ]	Human research participants
[x]	[ ]	Clinical data
[x]	[ ]	Dual use research of concern

Methods

n/a	Involved in the study	
[x]	[ ]	ChIP-seq
[x]	[ ]	Flow cytometry
[x]	[ ]	MRI-based neuroimaging

Antibodies

Antibodies used

The following primary antibodies were used in this study: guinea pig anti-MAP2 antibody 1:500 for immunofluorescence (IF) (Synaptic Systems, 188004), rabbit anti-MAP2 antibody 1:500 for IF (Synaptic Systems, 188002), mouse anti-αII spectrin antibody 1:400 for IF (Clone D8B7, Biolegend, 803201), mouse anti-αII spectrin antibody 1:200 for IF (Clone 3D7, Encor Biotechnology, MCA-3D7), rabbit anti-αII spectrin antibody 1:200 for IF (Encor Biotechnology, RPCA-aII-Spec), mouse anti-αII spectrin antibody 1:200 for IF (Clone AA6, EMD Millipore, MAB1622), mouse anti-βII spectrin antibody 1:200 for IF (Clone 42, BD Biosciences, 612563), mouse anti-dematin antibody 1:50 for IF (Clone 18, Santa Cruz Biotechnology, sc-135881), rabbit anti-coronin 2B antibody 1:200 for IF (Novus Biologicals, NBP 1-85567), mouse anti-tubulin antibody 1:100 for IF (Clone B7, Santa Cruz Biotechnology, sc-5286), rabbit anti-Tau antibody 1:500 for IF (Synaptic Systems, 314002), mouse anti-Kv1.2 channel antibody 1:200 for IF (Clone K14/16, Neuromab, 75-008), rabbit anti-neurofascin antibody 1:200 for IF (Clone A12/18, Neuromab, 75-172), rabbit anti-NrCAM 1:200 for IF (Abcam, ab24344), goat anti-CHL1 antibody 1:200 for IF (R&D systems, AF2147), rabbit anti-NCAM1 antibody 1:200 for IF (EMD Millipore, AB5032), mouse anti-ankyrin G antibody 1:100 for IF (Clone 463, Santa Cruz Biotechnology, sc-12719), mouse anti-bassoon antibody 1:400 for IF (Clone SAP7F407, Enzo, ADI-VAM-PS003-F), rabbit anti-homer antibody 1:500 for IF (Synaptic Systems, 160003), rabbit anti-L1CAM antibody 1:500 for Western blot (WB) (ABclonal, A8555), rat anti-L1CAM antibody 1:200 for IF (Clone 555, R&D Systems, MAB5674), rabbit anti-Myh10 (N-terminus) antibody 1:200 for IF (GeneTex, GTX133378), rabbit anti-Myh9 (N-terminus) antibody 1:200 for IF (GeneTex, GTX101751), rabbit anti-Myh10 (C-terminus) antibody 1:200 for IF (Biolegend, 909901), rabbit anti-Glutamate Receptor 2 & 3 antibody 1:200 for IF (EMD Millipore, AB1506), rabbit anti-GFP antibody 1:400 for IF (Thermo Fisher Scientific, A11122), rabbit anti-β-actin antibody 1:1000 for WB (Proteintech, 20536-1-AP).
The following secondary antibodies were used in this study: CF680-conjugated donkey anti-mouse IgG antibody 1:400 for IF (Biotium, 20819), Alexa-647-conjugated donkey anti-mouse IgG antibody 1:800 for IF (Jackson ImmunoResearch, 715-605-151), Alexa-647-

conjugated donkey anti-rabbit IgG antibody 1:800 for IF (Jackson ImmunoResearch, 711-605-152), Alexa-647-conjugated donkey anti-goat IgG antibody 1:800 for IF (Jackson ImmunoResearch 705-605-147), Alexa-647-conjugated donkey anti-rat IgG antibody 1:800 for IF (Jackson ImmunoResearch, 712-605-153), Cy3-conjugated donkey anti-rabbit IgG antibody 1:500 for IF (Jackson ImmunoResearch, 711-165-152), Cy3-conjugated donkey anti-guinea pig IgG antibody 1:800 for IF (Jackson ImmunoResearch, 706-165-148), Cy3-conjugated donkey anti-mouse IgG antibody 1:800 for IF (Jackson ImmunoResearch, 711-165-151). Alexa-488-conjugated donkey anti-guinea pig IgG antibody 1:800 for IF (Jackson ImmunoResearch, 706-545-148).

Validation

No validation was performed since all the antibodies used in this study were validated by the manufacturers and reported in the antibody datasheet, as indicated below:
-guinea pig anti-MAP2 antibody (Synaptic Systems, 188004): https://sysy.com/product/188004
-rabbit anti-MAP2 antibody (Synaptic Systems, 188002): https://sysy.com/product/188002
-mouse anti-αII spectrin antibody (Clone D8B7, Biolegend, 803201): https://www.biolegend.com/en-us/products/anti-alpha-ii-spectrin-antibody-10836
-mouse anti-αII spectrin antibody (Clone 3D7, Encor Biotechnology, MCA-3D7): https://encorbio.com/product/mca-3d7/
-rabbit anti-αII spectrin antibody (Encor Biotechnology, RPCA-aII-Spec): https://encorbio.com/product/rpca-aii-spec/
-mouse anti-αII spectrin antibody (Clone AA6, EMD Millipore, MAB1622): https://www.emdmillipore.com/US/en/product/Anti-Spectrin-alpha-chain-nonerythroid-Antibody-clone-AA6,MM_NF-MAB1622
-mouse anti-βII spectrin antibody (Clone 42, BD Biosciences, 612563): https://www.bdbiosciences.com/en-es/products/reagents/microscopy-imaging-reagents/immunofluorescence-reagents/purified-mouse-anti-spectrin-ii.612563
-mouse anti-dematin antibody (Clone 18, Santa Cruz Biotechnology, sc-135881): https://datasheets.scbt.com/sc-135881.pdf
-rabbit anti-coronin 2B antibody (Novus Biologicals, NBP 1-85567): https://www.novusbio.com/products/coronin-2b-antibody_nbp1-85567
-mouse anti-tubulin antibody (Clone B7, Santa Cruz Biotechnology, sc-5286): https://www.scbt.com/p/alpha-tubulin-antibody-b-7
-rabbit anti-Tau antibody (Synaptic Systems, 314002): https://sysy.com/product/314002
-mouse anti-Kv1.2 channel antibody (Clone K14/16, Neuromab, 75-008): https://www.antibodiesinc.com/products/anti-kv1-2-k-channel-antibody-k14-16-75-008?variant=12783172943931
-rabbit anti-neurofascin antibody (Clone A12/18, Neuromab, 75-172): https://www.antibodiesinc.com/products/anti-pan-neurofascin-extracellular-antibody-a12-18-75-172
-rabbit anti-NrCAM (Abcam, ab24344): https://www.abcam.com/nrcam-antibody-neuronal-marker-ab24344.html
-goat anti-CHL1 antibody (R&D systems, AF2147): https://www.rndsystems.com/products/mouse-chl-1-l1cam-2-antibody_af2147
-rabbit anti-NCAM1 antibody (EMD Millipore, AB5032): https://www.emdmillipore.com/US/en/product/Anti-Neural-Cell-Adhesion-Molecule-Antibody,MM_NF-AB5032?ReferrerURL=https%3A%2F%2Fwww.google.com%2F
-mouse anti-ankyrin G antibody (Clone 463, Santa Cruz Biotechnology, sc-12719): https://www.scbt.com/p/ankyrin-g-antibody-463
-mouse anti-bassoon antibody (Clone SAP7F407, Enzo, ADI-VAM-PS003-F): https://www.enzolifesciences.com/ADI-VAM-PS003/bassoon-monoclonal-antibody-sap7f407/
-rabbit anti-homer antibody (Synaptic Systems, 160003): https://sysy.com/product/160003
-rabbit anti-L1CAM antibody (ABclonal, A8555): https://abclonal.com/catalog-antibodies/KOValidatedL1CAMRabbitpAb/A8555
-rat anti-L1CAM antibody (Clone 555, R&D Systems, MAB5674): https://www.rndsystems.com/products/mouse-l1cam-antibody-555_mab5674
-rabbit anti-Myh10 (N-terminus) antibody (GeneTex, GTX133378): https://www.genetex.com/Product/Detail/MYH10-antibody/GTX133378
-rabbit anti-Myh9 (N-terminus) antibody (GeneTex, GTX101751): https://www.genetex.com/Product/Detail/MYH9-antibody-N1-N-term/GTX101751
-rabbit anti-Myh10 (C-terminus) antibody (Biolegend, 909901): https://www.biolegend.com/en-us/products/purified-non-muscle-myosin-heavy-chain-ii-b-antibody-11470
-rabbit anti-Glutamate Receptor 2 & 3 antibody (EMD Millipore, AB1506): https://www.emdmillipore.com/US/en/product/Anti-Glutamate-Receptor-2-3-Antibody,MM_NF-AB1506
-rabbit anti-GFP antibody (Thermo Fisher Scientific, A11122).
-rabbit anti-β-actin antibody (Proteintech, 20536-1-AP).

Eukaryotic cell lines

Policy information about cell lines

Cell line source(s)

U2OS (ATCC HTB-96) and HEK293T (ATCC CRL-3216)

Authentication

U2OS and HEK293T cells were not authenticated.

Mycoplasma contamination

U2OS and HEK293T cells were tested negative for mycoplasma contamination by source laboratory.

Commonly misidentified lines
(See ICLAC register)

No commonly misidentified cell lines were used.

Animals and other organisms

Policy information about studies involving animals; ARRIVE guidelines recommended for reporting animal research

Laboratory animals

The mouse lines used in this study include CFW mice (Strain code 024, Charles River Laboratories), a βII-Specflox/flox mouse line, a Nestin-Cre mouse line (Stock number 003771, The Jackson Laboratory), a whole body Tmod1 knockout mouse line that expresses a Tmod1 transgene only in the heart, a whole body Tmod2 knockout mouse line, and a whole body Dmtn knockout mouse line. Mice were housed at an ambient temperature of 19-23 °C with a humidity of 55% (± 10%) and 12-hr dark/light cycles. To make hippocampal neuronal cultures, hippocampi were isolated from mouse embryos at embryonic day 18 (for CFW, βII-Specflox/flox, and

	Nestin-Cre mice) or at postnatal day 0 (for Tmod1 knockout, Tmod2 knockout, and Dmtn Knockout mice). For imaging experiments, 4-6 embryos or newborn pups were used per condition; for co-IP experiments, 10-15 embryos were used per condition. Mouse pups were not sexed, and we expect approximately equal amounts of males and females. For proteomic analyses on adult mouse brains, female CFW mice at the age of 8-12 weeks were used.
Wild animals	No wild animals were used in the study.
Field-collected samples	No field collected samples were used in the study.
Ethics oversight	All experimental procedures were performed in accordance with the Guide for the Care and Use of Laboratory Animals of the National Institutes of Health. The protocol was approved by the Institutional Animal Care and Use Committee (IACUC) of Harvard University (protocol #10-16-3), Scripps Research Institute (protocol #08-0087), and Tufts University (protocol #B2021-159).

Note that full information on the approval of the study protocol must also be provided in the manuscript.
